# Case Report: Carotid–cavernous fistula due to aneurysmal rupture in a case of aortoaortitis with bilateral giant internal carotid artery aneurysms

**DOI:** 10.4103/0971-3026.57216

**Published:** 2009-11

**Authors:** Sandeep Sharma, Subhash Kumar, Arvind Nanda, Edmond Moses

**Affiliations:** Department of Neuroradiology, AIIMS, Ansari Nagar, New Delhi - 110 029, India

**Keywords:** Bilateral giant ICA aneurysms, carotid cavernous fistula, Takayasu arteritis

## Abstract

Takayasu aortoarteritis (TA) rarely affects the nervous system, but when it does, it usually manifests as cerebral ischemia or stroke. These strokes have mainly been attributed to stenotic extracranial vessels. Stenoses of intracranial vessels, although rare in TA, can occur due to either embolization into the vessel or because of the vasculitic process itself. Intracranial aneurysms are very rare in patients with TA. Bilateral cavernous internal carotid artery (ICA) aneurysms are rarer. They have been reported following radiation therapy and in association with fibromuscular dysplasia and juvenile Paget disease. Bilateral mycotic intracavernous aneurysms also occur. Bilateral giant cavernous ICA aneurysms with carotid-cavernous fistula (CCF) consequent to rupture into the cavernous sinus in a case of TA are extremely unusual. We report a case that fulfilled both American College of Rheumatology and European League against Rheumatology criteria for TA. The patient had bilateral cavernous sinus giant aneurysms and CCF because the right-sided aneurysm had ruptured and was leaking into the cavernous sinus.

## Introduction

Takayasu aortoarteritis (TA) is a large-vessel disease that causes aortic stenosis and/or stenoses of its major branches. A relatively rare, aneurysmal form of the disease is also known to occur.[1,2]

Carotid aneurysms are rare, but when they occur, they are predominantly extracranial.[1,3] Intracranial aneurysms are very rare. They usually occur in the vertebrobasilar system, associated with ipsilateral carotid stenosis, suggesting that hemodynamic stress may be the cause.[4] Bilateral cavernous internal carotid artery (ICA) aneurysms leading to carotid-cavernous fistula (CCF) have never been described in TA, to the best of our knowledge.

We present a case of TA in which bilateral giant intracavernous ICA aneurysms were present with rupture of the right aneurysm, leading to CCF. Other vessels were also involved in the disease process: bilateral renal artery stenosis, right mid-subclavian artery stenosis, right vertebral artery origin stenosis, right common iliac artery occlusion, and arch of aorta calcification.

## Case Report

**Figure 1(A–D):**
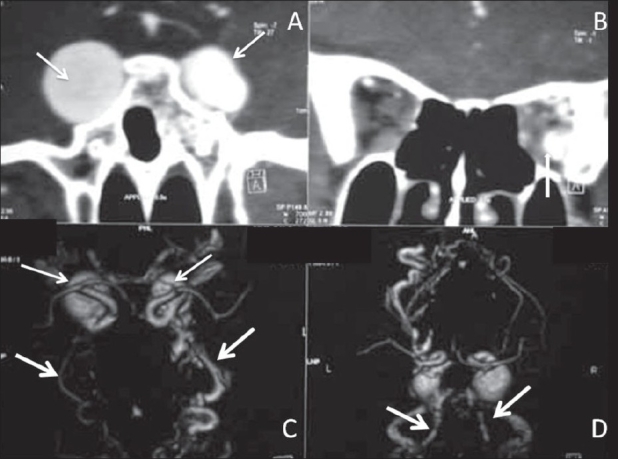
Computed tomography angiography (CTA) images. The coronal images (A, B) show bilateral cavernous internal carotid artery (ICA) giant aneurysms (arrows in A), with dilated ophthalmic veins (arrow in B). The volume rendering technique VRT images (C, D) show bilateral cavernous ICA aneurysms (arrows in C) as well as the early drainage through both the anterior ophthalmic veins (thick arrows in C) and the posterior petrous (thick arrows in D) system of veins

**Figure 2 (A, B):**
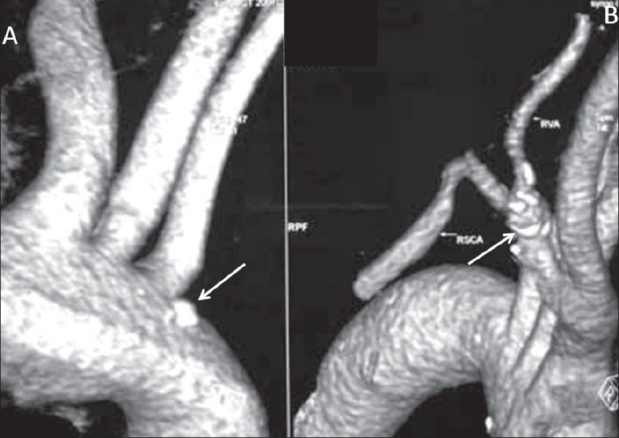
VRT images show a speck of calcification in the arch of the aorta (arrow in A) with narrowing and calcification of the right mid-subclavian artery (arrow in B), with involvement of the origin of the vertebral artery

**Figure 3 (A, B):**
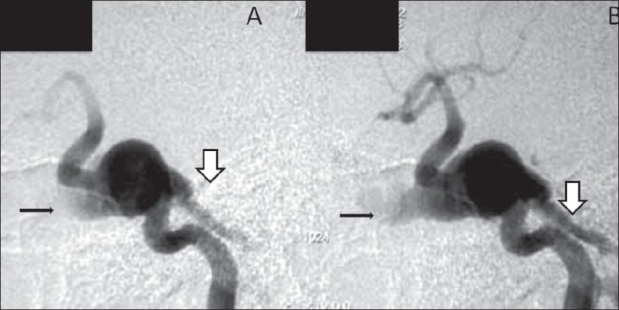
Lateral views of a digital subtraction angiogram study following a left internal carotid artery (ICA) injection show the cavernous ICA aneurysm with a carotico-cavernous fistula, leading to early filling of anterior (thin arrows) and posterior (thick arrows) veins

**Figure 4 (A, B):**
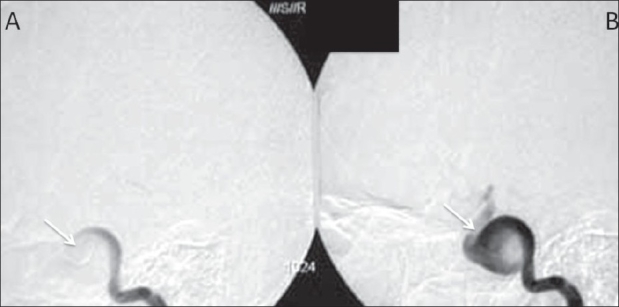
Lateral views of a digital subtraction angiogram study following a right internal carotid artery (ICA) injection show the cavernous ICA aneurysm (arrows)

A 17-year-old girl presented with a sudden onset of proptosis, pain, and chemosis of both eyes, the right eye being more severely affected. She also had subjective and objective tinnitus involving both ears. On examination, she had an elevated blood pressure, asymmetry of pulses, and an elevated erythrocyte sedimentation rate (ESR). USG of the abdomen revealed bilateral small kidneys. A color Doppler study revealed bilateral renal artery stenoses. A CT scan of the brain revealed enlarged, bulging cavernous sinuses and a large superior ophthalmic vein on the right side [Figure 1A, B]. Subtracted CT angiography (CTA) [Figures 1C, D and 2] was performed using a dual-source scanner (Definition, Siemens, Erlangen). Digital subtraction angiography (DSA) [Figures 3 and 4] showed bilateral giant cavernous ICA aneurysms. The right aneurysm [Figure 4] was shown to be leaking into the cavernous sinus, leading to early drainage through both superior ophthalmic veins and the petrous sinuses into the jugular veins. The patient's symptoms regressed over the next few days and spontaneous closure of the fistula was suspected. However, this could not be confirmed as the patient refused further investigations or treatment and was lost to follow-up.

## Discussion

According to the American College of Rheumatology (ACR), the presence of three of the following six features is diagnostic of TA[5]:

1. Age of onset before 40 years
2. Claudication
3. Decreased brachial artery pulse
4. Blood pressure difference of >10 mm Hg between the arms
5. Bruit over the subclavian arteries or aorta
6. Arteriogram abnormality

According to the more recent European League Against Rheumatism (EULAR)/Pediatric Rheumatology European Society consensus criteria, the diagnosis of TA in children requires the presence of angiographic abnormalities, on conventional, CT, or MRI angiography of the aorta or one of its major branches plus one or more of the following[6]:

1. Claudication or decreased peripheral artery pulses
2. Blood pressure difference >10 mm Hg between the arms
3. Bruits over the aorta or its major branches
4. Hypertension

On imaging, our patient had aortic arch calcification, calcification, and stenosis of the right subclavian artery also involving the vertebral artery origin, bilateral renal artery stenoses, and occlusion of the right common iliac artery, which was filling via collaterals. Clinically, she had asymmetry of pulses, hypertension, and an elevated ESR in addition to the cerebral symptoms. Thus, our patient fulfilled both ACR and EULAR criteria for the diagnosis of TA.

Cerebral ischemia or stroke is the usual central nervous system manifestation of TA, affecting about 20% of TA patients.[7,8] These strokes have mainly been attributed to stenotic extracranial vessels, but have increasingly also been recognized to be due to involvement of the intracranial vessels.[9,10]

Other intracranial manifestations of TA include the development of aneurysms. The development of cerebral aneurysms in patients with TA is often attributed to hemodynamic stress caused by increased flow in the affected arteries.[4] However, the intracranial arteries may be directly affected by TA and our case report shows a situation where the aneurysms symmetrically involved the cavernous carotid arteries.[9,11] There are about 20 reports of intracranial aneurysms in TA, in the literature.[12] These aneurysms have been reported in the paraclinoid, anterior communicating artery, middle cerebral artery M1 segment, anterior cerebral artery A1 segment, and the vertebro-basilar regions.[4,12] No case of intracavernous aneurysm has been reported in patients with TA till date.[4,12]
